# Morphometric and phytochemical characterization of chaura fruits (*Gaultheria pumila*): a native Chilean berry with commercial potential

**DOI:** 10.1186/0717-6287-47-26

**Published:** 2014-06-09

**Authors:** Evelyn Villagra, Carola Campos-Hernandez, Pablo Cáceres, Gustavo Cabrera, Yamilé Bernardo, Ariel Arencibia, Basilio Carrasco, Peter DS Caligari, José Pico, Rolando García-Gonzales

**Affiliations:** Departamento de Ciencias Forestales, Centro de Biotecnología de los Recursos Naturales, Universidad Católica del Maule, Campus San Miguel. Av. San Miguel 3605, casilla 617, Talca, Maule Region, Chile; Facultad de Agronomía, e Ingeniería Forestal, Pontificia Universidad Católica de Chile, Vicuña Mackenna 4860, Macul, Santiago, Chile; Instituto de Biología Vegetal y Biotecnología, Universidad de Talca, Avenida Lircay s/n, Talca, Chile

**Keywords:** *Gaultheria pumila*, Fruit morphometry, Fruit diversity, Pectin, Anthocyanins

## Abstract

**Background:**

For the first time, a morphometric characterization of chaura (*Gaultheria pumila*) fruits has been conducted between natural populations growing in the Villarrica National Park, Araucania Region, Chile. Chaura is a native Ericaceae from Chile that produces aromatic and tasty fruits which could be of agricultural interest.

**Results:**

To influence the decision for a further domestication of G*. pumila*, both the fruit sizes (indicator of productivity) and the nutritional properties of the fruits have been determined from different subpopulations. Samples were a total of 74 plants and 15 fruits per plant which were randomly harvested following its natural distribution around the Villarrica volcano. Altogether, fresh weight, shape, color, diameter in the pole and the equatorial dimensions were determined as phenotypic traits of the *G. pumila* fruits. Meanwhile the total soluble solids, anthocyanin and pectin contents were calculated as nutritional traits of the Chaura fruits. Results showed a high phenotypic diversity between the sampled population with three main fruit shapes and three predominant colors. The round shapes were the most abundant, whereas a significant correlation was found among fruit size with weight and color. The highest fresh weight (597.3 mg), pole diameter (7.1 mm) and equatorial diameter (6.5 mm) were estimated in the pink color fruits.

**Conclusions:**

The total amount of anthocyanin was higher in red fruits, while the maximum pectin content was obtained in the round white fruits. Overall results must pave the way for a further domestication and introduction of the Chaura species in the agro-productive system in Chile.

## Background

*Gaultheria pumila* is a native Chilean species from the Ericaceae family, commonly known as Chaura or Mutilla. *G. pumila* is a low bush that can reach up to 80 cm in height, depending on the environmental conditions. The species produces fleshy, flavored and aromatic fruits and inhabits the Andes mountains; its distribution ranges from the Region Metropolitana (33°26′ 16″ S; 70°39′ 01″ W) in the North to Region de Magallanes (53°9′ 45″ S; 70°55′ 21″ W) in the South [1]. Chaura berries in their natural habitat have been found for the wide variety of shapes and colors*.* Its natural habitat demonstrates extreme temperature variation, since in winter bushes are covered by snow for several weeks, and in summer the plants are exposed to high temperatures and high UV radiation. As a result of its adaptation, this species could be considered as an extremophile plant species because it is highly tolerant of harsh soil and environmental conditions [2]. In addition, Chaura leaves and fruits produce an intense aroma and flavor, which make it appealing for developing new food applications.

Fruit consumption is the main source of phenolics in the daily human diet, helping them to improve a large number of biological functions [3, 4]. The supply of natural phenolics compounds through the human diet has been associated to a reduction of heart and brain diseases as well as cancer [5, 6]. Polyphenols are a family of natural bioactive compounds with high antioxidant capacity that are capable of neutralizing and eliminating free radical species [7, 8]. It has been demonstrated that polyphenolic extracts from different sources are involved in the inhibition of low density lipoproteins (LDL), confirming their potential use as nutraceuticals with positive effects on human health [5, 9]. Ascorbic acid, tocopherols, tricotrienols, carotenoids, phenolic compounds and tannins can be considered among the most important plant derived antioxidants [10–12]. These compounds have been associated with anticancer activity, since they can assist complementary mechanisms of defense such as the induction of metabolizing enzymes and modulation of gene expression, cell proliferation and apoptosis [13].

Plant metabolites play a central role for protecting plants against herbivores and diseases; also they can attract pollinators and seed dispersers. Furthermore, secondary metabolites can also protect against abiotic stresses such as drought, temperature and radiation. Berry fruits, like many other species, are rich in flavonoids, phenolic acids, anthocyanin, vitamins, minerals and fiber [14, 15]. Polyphenol compounds produced in berries vary their concentration across different fruit tissues, and their total content could be influenced by several factors such as plant genotype, environmental and soil conditions in orchards, fruit maturity degree, harvest practices, and fruit storage conditions after harvest among others [16].

Chaura fruits show a resistant exocarp structure with a fleshy sweet mesocarp and many seeds. The appearance of the mesocarp suggests a high content of pectin in its structure. Pectins are complex heteropolysaccharides presented in the cell wall of land plants and fruits, providing consistency and mechanical resistance to vegetable tissues. The overall structure of these macromolecules encompasses homogalacturonan blocks (1,4-linked α-D-GalA units, which can be partially methylesterified), covalently linked to type I rhamnogalacturonan blocks (repeating disaccharide [→4)-α-D-GalA-(1 → 2)-α-L-Rha-(1→] units) bearing neutral sugar side-chains [17, 18]. Pectin is a worldwide food ingredient widely employed as gelling, emulsifying and stabilizing agents [19, 20]. It is a soluble fiber used in jams and jellies, fruit juices, fruit drink concentrates, desserts, baking fruit preparations, dairy and delicatessen products [21].

Also, pectin is important to human health, because it has many benefits, is a compound that appears to be able to inhibit cancer metastasis and primary tumor growth in multiple types of cancer in animals [22], and it can lower cholesterol in humans [23]. Pectins are industrially produced by diluted acid hot extraction from citrus and apples peels, respectively. Citrus and apple pectin content ranges from 15 to 30% (w/w) on dry weight base. Moreover, pectin content from other species has been studied, and high variability was found among them, for example, murta pectin content (30% w/w) [24], chickpeas (67% w/w) [25], Lobeira (33.6% w/w) [26], and sweet potatoes (10.24%) [27].

In Chaura, to the best of our knowledge there is no systematic information about morphometric characteristics of chaura fruits and no studies of chemical composition of the fruit has been done before. In addition, agricultural data about crop yield, growing habits, water and fertilizer needs, as well as fruit uses is almost nonexistent. Thus, Chaura remains as an unused but seemingly quite valuable Chilean genetic resource, and more studies exploring its potential as commercial fruit should be done.

In particular, this research was aimed to describe the fruit variation of natural populations of *G. pumila* (chaura) inhabiting the Villarrica National Park in Andeans mountains of South Chile. Morphometric characterization was performed by measuring color and fruit sizes, while phytochemical characterization is done by chemical analyzing peptin content, total anthocyanins and antioxidant activity of the fruits.

## Results

### Morphometric characterization and fruit size variation

**Figure 1:**
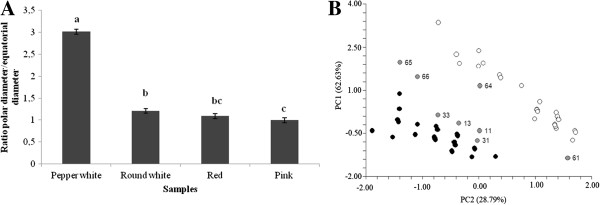
**Fruit variation in Chaura plants at Villarrica National Park. A)** ANOVA ratio of polar diameter/equatorial diameter per morphotypes of Chaura fruit. **B)** Principal components analysis based on fruit weight variation size and fruit color. Black dots, represent plants with pink and red fruits; white dots represents plants with white fruits. In grey dots, plants that did not grouped in any of the other two groups.

In order to know the main characteristics of Chaura fruits, a morphometric characterization was carried out; results are presented in Table 1. Results indicate that Chaura fruits show great phenotypic diversity and four different morphotypes can be clearly identified: pepper white, round white, red and pink. The red fruit showed the highest fresh weight value of 597.3 ± 274.4 mg, and it also had the highest value for the polar diameter (7.2 ± 2.2 mm) as well as the equatorial diameter (6.6 ± 1.7 mm). The white fruits had lower values for these three variables, except with polar diameter in pepper white. Thus, a comparative analysis of both weight and fruit size shows that there are significant differences between different morphotypes based on the two parameters analyzed, Table 1. Figure 1 shows the graphical analysis of mean from ratio polar diameter/equatorial diameter of Chaura morphotypes (p < 0.05).The Principal Component Analysis significantly demonstrated the relation among fruit weight and fruit color. As shown in Figure 1B, fruits with the highest equatorial size and weight were grouped in a single group of red and pink skin fruits. The smaller fruits with the lowest weight with white skin were grouped separately. Furthermore, eight plants did not belonged to any of the established groups. The effectiveness of the PCA analysis was probed with a discriminant analysis (Wilk’s lambda = 0.56; P < 0,00001), showing a 77.8% of consistency for classification based on fruit size and color.

**Table 1:** **Morphometry studies of** ***G. pumila*** **fruits collected in South Chile (La Araucania Region) near Villarica volcano (** ***n*** **= 156)**

Fruit	Fresh weight (mg)	Polar diameter (mm)	Equatorial diameter (mm)
Pepper white	223.0 ± 70.2 a	6.8 ± 2.1 a	2.5 ± 1.0 a
Round white	240.8 ± 103.9 a	4.2 ± 1.3 b	3.7 ± 1.4 b
Red	597.3 ± 274.4 b	7.2 ± 2.2 a	6.6 ± 1.7 c
Pink	456.7 ± 210.3 c	5.6 ± 2.1 c	5.5 ± 1.4 d

The evaluation of statistical significance was determined by ANOVA, followed by Tukey HSD test. Different letters represent statistical differences for each evaluated parameter (*p* < 0.05).

### Total solid content (°Brix)

The values obtained by the refractometer corresponded to 8.0 ± 1.4, 8.5 ± 0.7, 9.0 ± 1.2 and 10.0 ± 1.0° Brix for fruit round white, pink, pepper white and red, respectively. These values indicate the amount of soluble sugars present in fruits, so one degree °Brix is defined by one gram of sucrose in 100 g of solution. This represents the strength of solution as percentage by weight (% w/w) and reflects the state of maturity of the fruit at harvest [28].

### Anthocyanins content

**Figure 2:**
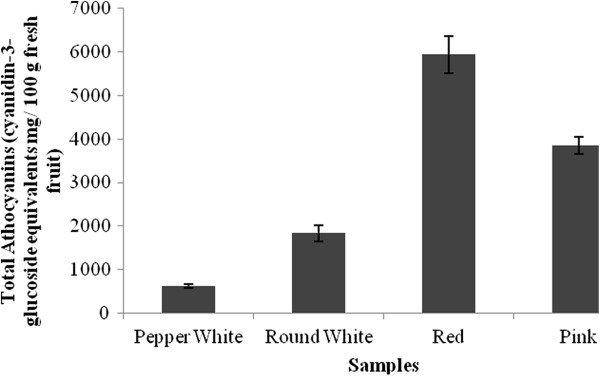
**Total Anthocyanins content by** ***G. pumila*** **morphotype.**

Figure 2 shows a significant difference in the content of anthocyanins by morphotype. The red fruit have higher values of anthocyanins with an average of 5,942 ± 422 mg monomeric anthocyanins per 100 g of sample. The pink fruits have an average of 3,854 ± 192 mg monomeric anthocyanins per 100 g of sample, while white fruits have the lowest anthocyanin content with an average of 626.2 ± 41 mg monomeric anthocyanins per 100 g of sample. Fruits shaped like a small white pepper are those with the lowest anthocyanin content.

### Pectin contents

**Figure 3:**
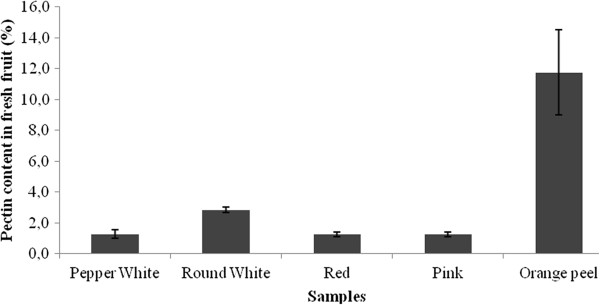
**Pectin content by** ***G. pumila*** **morphotype.** Orange peel was utilized as positive control.

Figure 3 shows the results of the pectin content in different Chaura fruits. For positive control, the pectin content in of orange peel (O), was used because pectin is extracted industrially from these peels. The results shown in Figure 3 are expressed in percentage of the fresh weight of fruit.

## Discussion

*G. pumila* is an example of underutilized genetic resource where phytochemical and morphometric features including fructification and organoleptic qualities have not been determined following scientific approaches. In this way, it was determined that the Chaura wild species could produce up to 0.5 kg of fresh fruits per plant, depending on the genotype and the environmental conditions. Regarding with the fruit weight, it can be seen that together polar and equatorial diameters contribute significant for the morphotypes pepper white and round white. Pepper white fruits have a long shape characteristic, while both red and pink morphotypes have a major size. Furthermore, both red and pink morphotypes have a higher weight in comparison with white fruits (Table 1). The Principal Component Analysis has shown a consistent grouping of the *G. pumilia* variability screened in this study.

Chaura fruit collected for this study showed lower Brix values than other fruits like cranberry, which at the time of harvest has values between 12 and 14° Brix [29]. The values obtained for Chaura vary depending on the color of the fruit: 8.5° Brix for pink fruit, 8.0° Brix for round white fruit and 9.0° Brix for pepper white fruit, while the red fruits, showed the highest value at 10.0° Brix. Despite it is necessary to collect and characterize the weather and soil conditions near the Villarrica Vulcano, where these three populations were collected, that ° Brix variation could be explained by the influence of the microclimatic conditions for each population. In general, the Villarrica National Park area near the volcano is characterized by two climatic zones. The first is more temperate: warm during the day in summer and drier, and during winter light frost or snowing occurs. Temperatures ranging between 4.0°C and 23.0°C, averaging an annual temperature of 11.5°C. In this area, the forest is very fragmented and large areas are exposed directly to sunlight. The first sampled population (El Playón) and the third sampled population (Cuesta Amarilla) are placed in this zone. The second climatic zone where the third population in this study (Centro Sky) was sampled is characterized by abundant snow and rainfall, which can reach 2,045 mm and 3,000 mm per year, largely concentrated in the months of May and August. In this zone Chaura grows mainly under the forest in a very humid environment and with no exposure to the direct sunlight radiation. Also, this population is placed in a higher altitude than the two other populations.

Another important factor could be the topography, characterized by gorges and high peaks (2,847 meters above sea level (masl)), these differences in sampling zones may influence on the ecological plasticity of the species against different environmental conditions [30] so that at harvest, the fruits do not have a homogeneous maturity. Therefore, the time to harvest the fruits, together with the microclimate generated in different sectors of sampling could also be influencing the development of the various morphotypes, as has been found before for other plant species [31–33]. However, it is remarkably necessary to correlate the phenotypic variation inside the whole studied area with the climatic conditions and for instance it is necessary to characterize in deep the climatic values for the whole area.

The fruit with intense red pigmentation had the highest content of monomeric anthocyanins, which was nine times higher than in the white fruit values. The pigmentation is directly related to the anthocyanins content because they give the red, purple and blue to the leaves, flowers and fruits [34]. Anthocyanins of berries have been reported to have potential health benefits [35]. The anthocyanin cyanidin 3-glucoside is the main active compound that has demonstrated bioactivity against carbon tetrachloride, avoiding lipid peroxidation, a process that eventually causes cell membrane to lose its physicochemical properties, culminating in cell death [36]. According to Prior (1998), anthocyanins in blueberries are mainly concentrated in the epicarp of the berry, but the amount of this chemical would increase depending on the area/volume ratio, that is related with fruit maturity. Several varieties of the genus *Vaccinium* have shown values between 61.8-235 mg/100 g [35]; whereas in caneberries, values of anthocyanins have been established from 65 mg/100 g in red raspberry to 589 mg/100 g in black raspberries [6]. Strawberries have shown 10 times higher values than raspberry with 5400 mg/100 g [37]; all these cases used cyanidin 3-glucoside as the standard. Comparing these data with the results of this research, which yielded concentrations from 626.2 mg/100 g (morphotype white pepper) to 5942 mg/100 g (morphotype red), demonstrated that the fruits of Chaura, naturally high in anthocyanins, exceeded the anthocyanins values of non-native berries.

Using a standard extraction method, the pectin content from Chaura fruits and orange peel were compared. The results show that the fruits of Chaura have an important content of pectins, reaching about 3% of fresh weight pectins compared to 12% for the pectin content in the orange peel. Furthermore, no significant difference was found between the pectin content of the four morphotypes of Chaura. Although the percentage of pectin in the Chaura fruit is quantitatively lower than in the orange peel, it must be noted that the pectin content was obtained from the full fruit of the Chaura, in contrast with what happens in orange peel. The pectin in orange peel only represents about 12% of fresh weight, and the peel is not considered edible. Assuming that pectins are capable of providing health benefits in terms of lowering cholesterol [23], to use Chaura fruits alone could improve the organoleptic characteristics when used as a functional food additive [19, 20]. Thus these fruits have high nutraceutical properties in addition to the antioxidant properties that might arise from the content of anthocyanins.

## Conclusions

This paper deals with the first morphometric analysis and phytochemical study in *Gaultheria pumila* (Chaura) fruits from a wild species from Chile commonly known as Chaura. It was found that there is significant morphometric difference in the fruit depending on fruit color and size. Moreover, a significant difference was found in the fresh weight between unpigmented and pigmented fruit, with pink fruit showing higher fresh weight, and larger polar and equatorial diameters. In relation to Chaura phytochemistry, burgundy colored fruits had a higher concentration of anthocyanins, comparable to other berries with commercial interest, such as blueberries. In addition, high pectin content gives additional commercial value for its intrinsic ability to form gels. Both anthocyanins and pectins have proven beneficial to health. Finally, there is little information regarding native berries and bioactive compounds, which is why *G. pumila* is presented as an underutilized genetic resource, with phytochemicals and morphometric features of great interest, good fruit, and attractive organoleptic characteristics. These preliminary results could open the interest of farmers and the food industry in this species and will be precedent for future domestication and introduction of the species in the agro-productive system in Chile and the rest of the world.

## Methods

### Plant material

**Figure 4:**
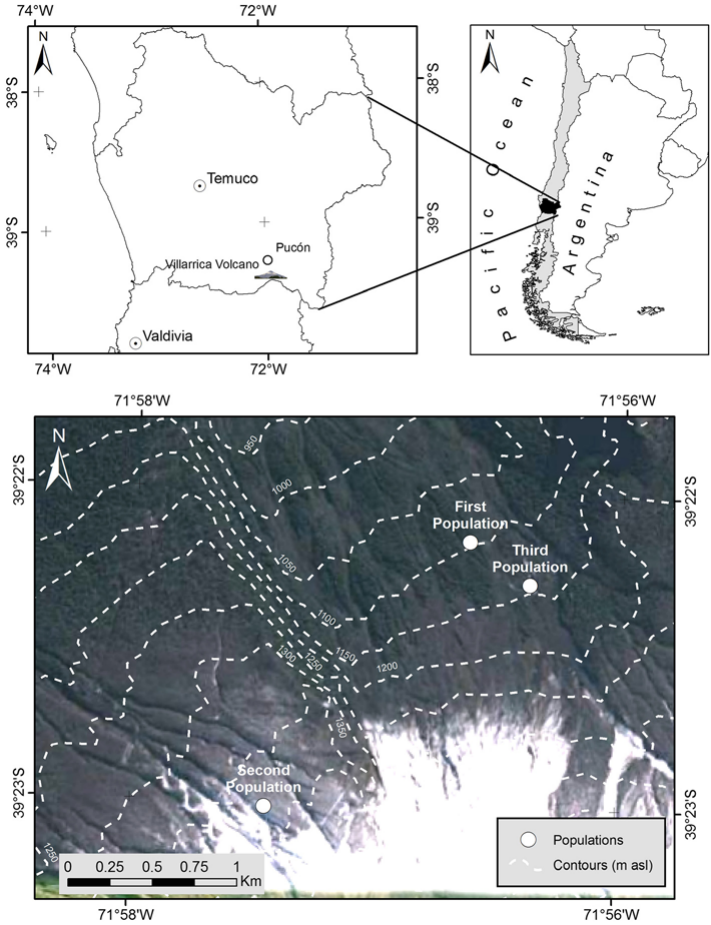
**The population distribution of** ***G. pumila.*** Three different wild populations in the Villarrica National Park, Región de la Araucanía, Chile. First population growing the sector locally known as El playón; Second population growing in the sector known as Centro Sky; Third population growing in the sector known as Cuesta Amarilla.

**Figure 5:**
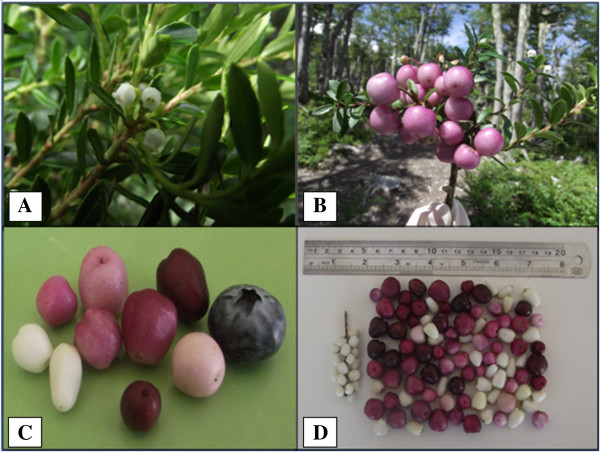
**Flowering and** ***G. pumila*** **diversity. A)** Flowering and inflorescence of Chaura. **B)** Globe shape and pink color fruits. **C)** Comparison of different morphotypes Chaura fruits, with a blueberry commercial gauge. **D)** Fructification and phenotypic diversity Chaura fruits.

Fruits were sampled from 74 plants coming from three different wild populations grown in the area of Villarrica volcano, this volcano is placed in the Villarrica National Park, Región de la Araucanía, Chile. The coordinates of the first population (named as El Playón) are 39° 22′ 21″ S; 71° 56′ 4″ W, of the second population (named as Centro Sky) are 39° 23′ 13″ S; 71° 57′ 36″ W, and the third population (named as Cuesta Amarilla) are 39° 22′ 29″ S; 71° 56′ 3″ W (Figures 4 and 5). Fruits from plants of each geographic population were randomly harvested in February-March during the summer of 2011. Harvested fruits were stored in a cooler containing ice at 4°C, approximately, until their transport to the controlled laboratory conditions. Once in the lab, the fruits were stored in a refrigerator at 4°C, until the analysis was performed.

### Fruit characterization

Morphometric characterization was conducted on fifteen fresh fruits of different shapes (pepper, full cone, and round shape), color (white, red or pink), and diameter (both pole and equator). Shape and color were evaluated visually, and a micrometer caliper (Tornado tools, Taiwan) was used to measure diameter in millimeters (mm).

### Total soluble solid content (TSS)

The total soluble solid of the fruits includes organic acids, carbohydrates (sugars), and amino acids [38]. In order to determine the (TSS) of fruit samples, twenty fresh fruits were measured with a digital refractometer (Atago Master M, Atago Co Ltd., Japan) with a scale of 0–32° Brix was used. Results were expressed as degree Brix (°Brix).

### Totals anthocyanins extraction

Fresh chaura fruits (2.5 g) were mixed with acidic ethanol (pH 4) at 1:10 (w/v) ratio. The mixture was sonicated for 15 min, and shaken for 60 min at room temperature. Then, the mixture was centrifuged (Boeco, U-320R, Alemania) at 4°C for 20 min at 5000 *g*. Supernatants were filtered through a funnel with glass wool, which had been washed with 3–4 mL of solvent. The extraction was repeated three times, and supernatants were pooled and stored at −20°C for further analysis. Three replications per sample were carried out.

### Total anthocyanin content (TAC)

The pH differential spectrophotometric method [39] was employed for quantifying the TAC in samples, using cyanidin-3-glucoside as the standard. Briefly, 200 μL of properly diluted water extract samples were mixed with 1.8 mL of either 25 mM HCl/KCl, pH 1.0 and 0.4 M acetic acid/sodium acetate, pH 4.5 solutions. The absorbance of these solutions was measured at 520 nm and 700 nm (UV mini-1240, SHIMADZU, Japan) TAC was estimated according to the following equation:

A = Absorbance of the sample

MW = Molecular weight of the standard (449.2 g mol-1)

V = Volume of the sample

Dil: Dilution of the sample

ϵ = Molar extinction coefficient of the standard (26 900 L cm-1 mol-1)

l = Cuvette depth

m = mass of fresh fruits

To perform this analysis 2.5 g of fresh fruit sampled from different plants of each fruit type were analyzed. Three replications per sample were carried out.

### Pectin extraction

5 g of frozen fruit were ground with a crusher in 50 mL water at pH 2.0, and the mixture was placed in an ultrasound bath at 80°C for 30 min. The solution was filtered hot, and then 50 mL of ethanol were added. Once the clot formed, the solution was centrifuged at 4000 rpm for 20 min, after which time the supernatant was discarded. The pectins obtained were lyophilized to determine the weight of the extract [26, 40].

### Statistical analysis

Descriptive statistics (mean and standard deviation), ANOVA and Turkey test (HSD) were performed using the commercial software package, Statgraphics centurion XV, version 15.01.03 (Statpoint technologies, Warrenton, VA, USA), level of significance was set at *p* < 0.05. A Principal Component Analysis was performed for fruit shape and color and grouping was confirmed by using a discriminant analysis (Wilk’s lambda; P < 0.00001). Graphical representations of results were performed using the software Microsoft Excel from Microsoft Office 2007.
